# 2-Chlorophenyl Zinc Bromide: A Convenient Nucleophile for the Mannich-Related Multicomponent Synthesis of Clopidogrel and Ticlopidine

**DOI:** 10.3390/molecules15118144

**Published:** 2010-11-11

**Authors:** Isabelle Aillaud, Caroline Haurena, Erwan Le Gall, Thierry Martens, Gino Ricci

**Affiliations:** 1 Électrochimie et Synthèse Organique, Institut de Chimie et des Matériaux Paris Est, ICMPE, UMR 7182 CNRS - Université Paris-Est Créteil Val-de-Marne, 2-8 rue Henri Dunant, 94320 Thiais, France; E-Mails: iaillaud@cpe.fr (I.A.); haurena@glvt-cnrs.fr (C.H.); martens@glvt-cnrs.fr (T.M.); 2 Sanofi-Aventis, Process Development, 45 Chemin de Mételine, BP 15, 04201 Sisteron cedex, France; E-Mail: gino.ricci@sanofi-aventis.com (G.R.)

**Keywords:** multicomponent reactions, organozinc reagents, amino acids, cobalt, zinc

## Abstract

A methodological study devoted to the Mannich-like multicomponent synthesis of the antiplatelet agent (±)-clopidogrel (**7**) and the ethyl ester analogue **6** is described. The process involves the formation of 2-chlorophenyl zinc bromide (**2**) and its subsequent reaction with an alkyl glyoxylate and 4,5,6,7-tetrahydrothieno[3,2-c]pyridine (**3**). We demonstrate that the organozinc reagent **2** also constitutes a very convenient nucleophile for the multicomponent synthesis of the benzylamine core of ticlopidine (**9**).

## Introduction 

**Scheme 1:**
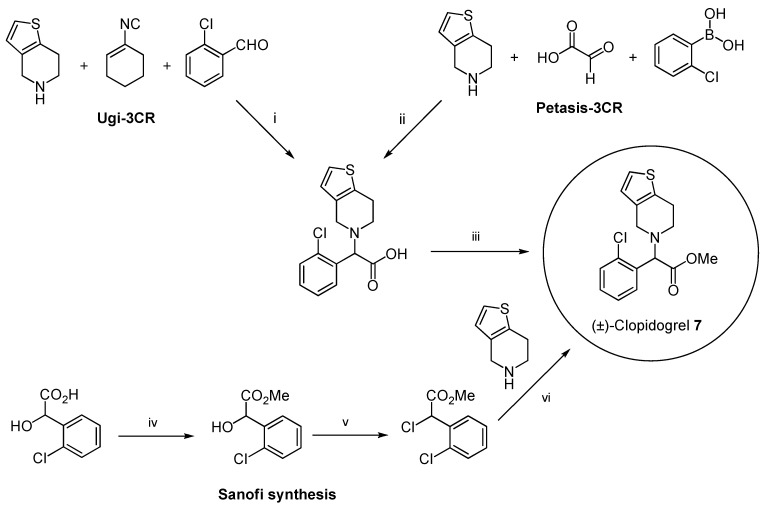
Some representative examples of (±)-clopidogrel syntheses.

The development of new or improved methods for the straightforward preparation of pharmaceuticals has been the subject of great interest over the last few years, e.g. for the production of generics. In this context, the synthesis of the antiplatelet agent clopidogrel, which was the second top selling drug in the mid 2000s, has been extensively investigated [1]. Curiously, in spite of their high synthetic interest, the use of multicomponent procedures [2,3,4,5,6,7,8,9,10,11] for the synthesis of (±)-clopidogrel (**7**) has been only scarcely reported. For instance, as this compound can be regarded as the ester derivative of a non-natural *α*-amino acid, some well-established multicomponent procedures allowing the formation of this core unit have been used as the key step of its preparation. Accordingly, the synthesis of **7** has been reported recently by Kalinski and co-workers [12], either by using the Petasis [13,14,15] or the Ugi [16,17] reaction. Compared to the original patented Sanofi multi-step synthesis of clopidogrel [18], the use of these multicomponent procedures allows the preparation of **7** both in a more straightforward manner and with better overall yields (Scheme 1).

In previous papers, we reported a straightforward synthesis of various nitrogen-containing compounds by using a Mannich-type multicomponent reaction between aromatic organozinc reagents, amines, and carbonyl derivatives [19,20]. It was noticed that a wide range of structural units are accessible using this methodology, but we did not investigate the potential use of this procedure for the preparation of pharmaceuticals. Consequently, we intended herein to apply this methodology to the synthesis of some famous drugs such as the antiplatelet agents (±)-clopidogrel (**7**) and ticlopidine (**9**).

## Results and Discussion

**Scheme 2:**
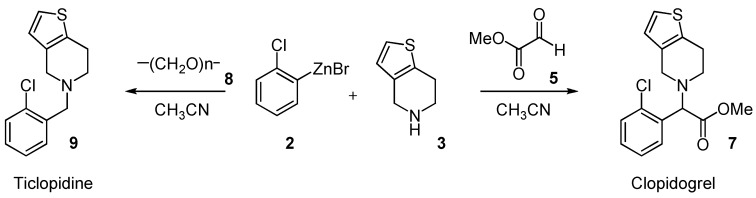
Projected multicomponent synthesis of clopidogrel and ticlopidine.

It was anticipated that both targets should be accessible through a three-component reaction between 2-chlorophenylzinc bromide (**2**), 4,5,6,7-tetrahydrothieno[3,2*-c*]pyridine (**3**), and methyl glyoxylate (**5**) [for the synthesis of (±)-clopidogrel (**7**)] or paraformaldehyde (**8**) [for the synthesis of ticlopidine (**9**)]. The projected syntheses are outlined in Scheme 2.

**Scheme 3:**
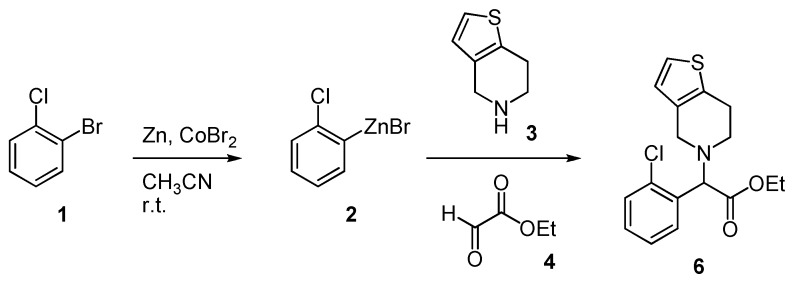
Synthesis of the clopidogrel ethyl ester analogue **6**.

As a starting point of the study, we turned our attention to the synthesis of methyl 2-(2-chlorophenyl)-2-(6,7-dihydrothieno[3,2-*c*]pyridin-5(*4H*)-yl)acetate [(±)-clopidogrel (**7**)]. As indicated above, with our multicomponent process in hands, the synthesis of **7** might be achievable in one step by using methyl glyoxylate as the carbonyl-containing compound. However, due to its ready commercial availability, we envisaged carrying out an optimization study starting from ethyl glyoxylate [21] instead of methyl glyoxylate. It was anticipated that methyl glyoxylate should possess a similar reactivity and therefore, we planed to make the use of this compound only after the optimization step. Alternatively, (±)-clopidogrel (**7**) should also be reachable by a simple transesterification of its ethyl ester analogue **6**. The synthesis of this latter was envisaged as follows: 2-bromochlorobenzene (**1**) would be converted into 2-chlorophenyl zinc bromide (**2**), which would be allowed to react with 4,5,6,7-tetrahydrothieno[3,2*-c*]pyridine (**3**) and ethyl glyoxylate (**4**) to furnish the clopidogrel ethyl ester analogue **6** (Scheme 3).

We then tried to define accurate reaction conditions for the preparation of **6** by assessing a broad range of reaction parameters like the amounts of reagents, the medium temperature, the reaction scale, the concentration of reagents, *etc*. In a first series of experiments, we turned our attention to evaluating the effect of different amounts of organozinc reagent on the three-component coupling efficiency. To this end, an initial set of conditions had to be defined. In a previous study dealing with the multicomponent synthesis of α-amino esters, it was noticed that a range of arylzinc halides react efficiently at room temperature with amines and ethyl glyoxylate, provided that the latter is used in excess. Thus, in a typical experiment, 2-chlorophenylzinc bromide (**2**) was prepared in acetonitrile from the corresponding aryl bromide **1** according to the general procedure, closely related to the original procedure of Gosmini and coworkers [22-24]. Various amounts [25] of this organometallic reagent were then allowed to react with the amine **3** (1 mmol) in the presence of 2.5 equiv. [26] of ethyl glyoxylate (**4**) [27], for 0.5-1 h at room temperature [28]. Results are presented in Table 1.

These experiments revealed that at least 2.5 equiv. of **2** are required for the reaction to start (Table 1, entry 3). The minimum value for the completion of the reaction is 3 equiv. of the organozinc (Table 1, entry 4). In that case, a 100% conversion is observed after only 30 min at room temperature, along with a very good isolated yield (82 %) [29]. It is worth noting that beyond 3 equiv., the reaction proceeds quantitatively (GC monitoring, Table 1, entries 5 and 6). However, in a general manner, since comparable isolated yields should be obtained when quantitative GC yields are observed, it was not intended to systematically extract the coupling products.

In another set of experiments, we examined the influence of amount of ethyl glyoxylate on the three-component coupling. Having previously demonstrated that the organozinc species **2** has to be used in sufficient excess to obtain efficient couplings, the following experiments were realized on a 1 mmol scale (amine **3**) with a large excess of **2** in the reaction medium. Results are summarized in Table 2.

These experiments indicate that a quantitative consumption of the amine **3** (and a 78 % yield) could be achieved, after 30 min reaction time, starting from 2 equiv. of ethyl glyoxylate (Table 2, entry 2). It can be noted that the use of larger amounts of ethyl glyoxylate gave similar results (Table 2, entries 3 and 4). Taken together, the results presented in Table 1 and Table 2 indicate that both reagents other than the amine **3** have to be used in excess. Consequently, the amine might be used as the limiting reagent and a rise of its amount should result in the decrease of the reaction efficiency. This hypothesis could be verified as following: starting from the optimum conditions defined in entry 2 of Table 2, the amine amount was increased from 1 to 2 mmol, resulting in the important decrease of its consumption from 100 to 15 %, even after 2 h at room temperature. Starting from the optimized conditions (Table 2, entry 2), we next envisaged to assess the influence of the reaction temperature. Results are presented in Table 3.

It can be noted that while the reaction substrates do not undergo coupling at 0 °C (Table 3, entry 1), a moderate rise of the temperature to 25°C already provides efficient conversions in short reaction times (Table 3, entry 2), while at 60°C, the reaction goes to completion in less than 20 min (Table 3, entry 3).

All previously-described experiments were realized on a 1 mmol scale (of amine **3**). Thus, we next tried to change the reaction scale by using increased amounts of the amine **3** in the presence of 2 equiv. of glyoxylate **4** and at least 3 equiv. of the organozinc **2**. Results are presented in Table 4.

A scale-up of the reaction appears feasible. Indeed, similar conversions and yields are obtained with an amine amount ranging from 1 to 30 mmol. However, for technical reasons, we did not try to raise the amine amount above this latter quantity.

In a second part of the study, we tried to extend the method to the synthesis of (±)-clopidogrel **7**, by simply exchanging ethyl glyoxylate (**4**) for methyl glyoxylate (**5**). Unfortunately, preliminary experiments realized using (neat) methyl glyoxylate resulted in failure as no conversion of the amine **3** was detected, even after several hours under heating. Two hypotheses were envisaged to explain these failures: methyl glyoxylate is supplied as a mixture of the aldehyde and the corresponding hydrate. In that case, a part of the organozinc might be lost instantly through acid-base reaction with the hydrate. However, the chromatographic monitoring of a test reaction did not reveal a significant loss of the organozinc reagent upon mixing, thus allowing us to discard this hypothesis. Another possibility lies in the lack of reactivity of the polymeric form(s) of methyl glyoxylate. The well-known tendency of this compound to polymerize rapidly, following a strong exothermic reaction, may account for a difficult reverse reaction. We thus envisaged a depolymerization step by applying a vigorous heating to **5** prior to the three-component coupling. Unfortunately, conventional heating using an oil bath did not allow obtaining **7**, even in trace amounts. 

**Scheme 4:**
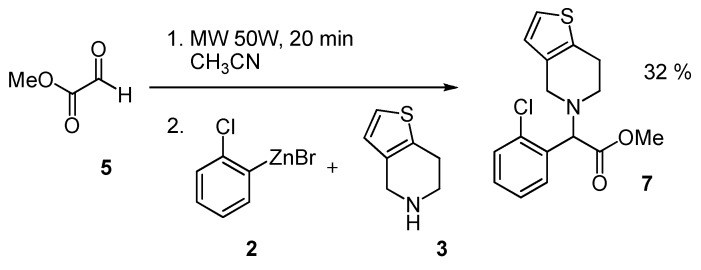
Synthesis of (±)-clopidogrel (**7**).

We then turned our attention to the microwave (MW) activation of the reaction. Thus, in a preliminary step, methyl glyoxylate was subjected to a focused microwave irradiation (50 W for 20 min). After addition of both other reagents **2** and **3**, a 40 % conversion and a 32 % isolated yield were obtained (Scheme 4).

Even though this result could not be further improved [30], it indicates that the synthesis of (±)-clopidogrel (**7**) from methyl glyoxylate (**5**), 4,5,6,7-tetrahydrothieno[3,2-*c*]pyridine (**3**) and 2-chlorophenyl zinc bromide (**2**) following a Mannich-like multicomponent procedure is clearly possible.

We then tried to obtain (±)-clopidogrel (**7**) through transesterification of its ethyl analogue **6**. In this purpose, some common methods were evaluated. Results are summarized in Table 5. The first point to note concerns the overall difficulty of transesterifying **6**, probably due to the presence of a nitrogen atom close to the ester function. Consequently, an acid catalysis of the reaction is not efficient (Table 5, entry 1) and the acid has to be used in excess (Table 5, entry 2). This is also the case with iodine (alone or in conjunction with indium) which must be used in excess to obtain a significant conversion of the starting amine (Table 5, entries 4 and 5). Best results are obtained with methylate ions as a transesterification agent, with a quasi-quantitative conversion of **6** into (±)-clopidogrel (**7**), after a 30 minutes heating period.

**Scheme 5:**
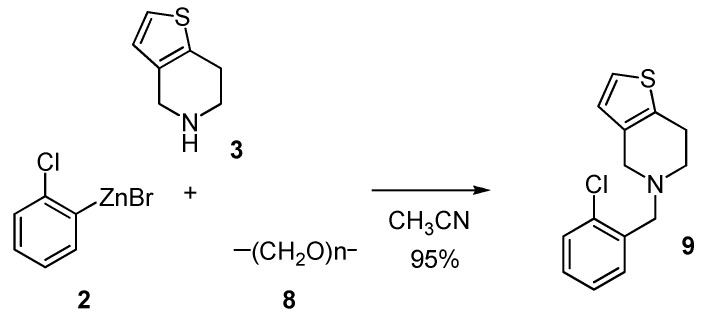
Synthesis of ticlopidine **9**.

In a third part of the study, we attempted to synthesize ticlopidine (**9**) by a three-component reaction between 2-chlorophenylzinc bromide (**2**), paraformaldehyde (**8**) and 4,5,6,7-tetrahydrothieno[3,2-*c*]pyridine (**3**). Thus, the organozinc **2** (> 3 equiv.) was allowed to react with the amine **3** and paraformaldehyde (**8**) (1.8 equiv., depolymerized prior to use by a 2 h heating period at 60 °C) for 1 h at 60 °C. This experiment resulted in the formation of the expected target in 95 % yield, thus pointing-out the relevance of this strategy for the synthesis of the benzylamine backbone (Scheme 5).

## Experimental 

### General

Solvents and reagents were purchased from commercial suppliers and used without further purification. All reactions were monitored by gas chromatography (GC) using a Varian 3400 chromatograph with a capillary DB1 column (l = 5 m, Ø = 0.32 mm, df = 0.4 µm). Reactions involving microwave heating were realized using a CEM Discover system. NMR spectra were recorded in CDCl_3_, at 400 MHz (^1^H) and 100 MHz (^13^C) on a Bruker Avance II 400 spectrometer. Chemical shifts (*δ*) are reported in parts per million (ppm) relative to the residual solvent signal. Coupling constant values (*J*) are given in Hertz (Hz) and refer to apparent multiplicities, indicated as follows: s (singlet); d (doublet); t (triplet); q (quartet); m (multiplet); dd (doublet of doublets). ESI^+^ mass spectra were recorded on a Trace 200 chromatograph with a capillary column CPSIL5CB/MS (l = 25 m, Ø = 0.25 mm, df = 0.12 µm). Characterization data of the coupling products matched the data reported in the literature [31-33].

*General procedure for the synthesis of 2-chlorophenyzinc bromide* (**2**)**.** A 25 mL round-bottom flask was flushed with argon and charged with acetonitrile (7 mL). Dodecane (0.04 mL), zinc dust (1.2 g, 18 mmol), trifluoroacetic acid (0.04 mL) and 1,2-dibromoethane (0.1 mL) were added and the solution was heated under vigorous stirring until gas was evolved. Heating was stopped and the solution allowed to cool for 15 min. 2-Bromochlorobenzene (**1**, 0.7 mL, 6 mmol) and cobalt bromide (0.13 g, 0.6 mmol) were added to the mixture which was stirred at ambient temperature for additional 30 min, resulting in the formation of 2-cholorophenyl zinc bromide (**2**) in ~75 % yield [34].

*Multicomponent synthesis of ethyl 2-(2-chlorophenyl)-2-(6,7-dihydrothieno[3,2-c]pyridin-5(4H)-yl)acetate* (**6**). To a 25 mL round-bottom flask, flushed with argon, was added acetonitrile (2 mL) and ethyl glyoxylate (**4**, ~50 % w/w solution in toluene, 0.4 mL, 2 mmol). The resulting solution was heated at 60 °C for 20 minutes then cooled down to room temperature. The amine **3** (0.14 g) and the solution of organozinc **2** were successively added and the mixture was stirred for 30 minutes. A saturated ammonium chloride solution (30 mL) was added and the organic products were extracted with dichloromethane (2 × 50 mL). The organic fraction was washed with water (30 mL), dried over MgSO_4_ then concentrated under reduced pressure. The remaining crude oil was purified by silica gel chromatography using a 89/9/2 pentane/Et_2_O/NEt_3_ mixture as an eluant to furnish **6** as a pale yellow oil (0.262 g, 78 %). ^1^H-NMR: *δ* 7.71 (dd, *J* = 7.6, *J* = 2.0 Hz, 1H), 7.41 (dd, *J* = 7.6, *J* = 2.0 Hz, 1H), 7.28 (dd, *J* = 5.6, *J* = 1.6 Hz, 1H), 7.28-7.25 (m, 1H), 7.06 (d, *J* = 5.1 Hz, 1H), 6.67 (d, *J* = 5,1 Hz, 1H), 4.88 (s, 1H), 4.22-4.14 (m, 2H), 3.75 (d, *J* = 14.1 Hz, 1H), 3.63 (d, *J* = 14.1 Hz, 1H), 2.88-2.89 (m, 4H), 1.22 (t, *J* = 7.1 Hz, 3H); ^13^C-NMR: *δ* 171.0, 134.8, 134.1, 133.5, 133.4, 130.0, 129.9, 129.4, 127.2, 125.4, 122.8, 68.1, 61.2, 50.7, 48.4, 25.6, 14.3; MS (ESI^+^): m/z = 335 (1), 264 (37), 263 (18), 262 (100), 207 (10), 154 (14), 152 (46), 138 (14), 125 (22).

*Multicomponent synthesis of methyl 2-(2-chlorophenyl)-2-(6,7-dihydrothieno[3,2-c]pyridin-5(4H)-yl)acetate* [(±)-*clopidogrel* (**7**)]. To a 25 mL round-bottom flask, flushed with argon, was added acetonitrile (2 mL) and methyl glyoxylate **5** (0.18 g, 2 mmol). The flask was placed in the microwave oven and heated for 30 min at 50 W. The amine **3** (0.14 g) was added and the resulting solution was submitted to the action of an additional 50 W over 10 min program. The solution of organozinc **2** was added to the flask and the resulting mixture was heated for additional 30 minutes at 50 W. A saturated ammonium chloride solution (30 mL) was added and the organic products were extracted with dichloromethane (2 × ×50 mL). The organic fraction was washed with water (30 mL), dried over MgSO_4_ then concentrated under reduced pressure. The remaining crude oil was purified by silica gel chromatography using a 89/9/2 pentane/Et_2_O/NEt_3_ mixture as an eluant to furnish **7** as a pale yellow oil (0.103 g, 32 %). ^1^H-NMR: *δ* 7.70 (dd, *J* = 8.0, *J* = 1.0 Hz, 1H), 7.41 (dd, *J* = 8.0, *J* = 1.6 Hz, 1H), 7.25-7.15 (m, 2H), 7.00 (d, *J* = 4.0 Hz, 1H), 6.61 (d, *J* = 4.0 Hz, 1H), 4.85 (s, 1H), 3.70 (d, *J* = 12.0 Hz, 1H), 3.64 (s, 3H), 3.57 (d, *J* = 12.0 Hz, 1H), 2.82 (s, 4H); ^13^C-NMR: *δ* 171.3, 134.7, 133.7, 133.2, 130.0, 129.8, 129.5, 127.2, 125.3, 122.8, 67.8, 52.2, 50.7, 48.3, 25.5; MS (ESI^+^): m/z = 320 (1), 264 (41), 263 (18), 262 (100), 154 (14), 152 (44), 138 (16), 125 (20).

*Synthesis of methyl 2-(2-chlorophenyl)-2-(6,7-dihydrothieno[3,2-c]pyridin-5(4H)-yl)acetate,* [(±)-*clopidogrel* (**7**)] *from* **6**: A 10 mL round-bottom flask was flushed with argon and charged with methanol (2 mL). Small portions of sodium (0.2 g, 8.7 mmol) were added at 0 °C and the mixture was stirred until complete dissolution. The α-amino ester **6** (0.68 g, 2 mmol) was added and the resulting solution was refluxed for 30 minutes then cooled down to ambient temperature. Water (10 mL) was added and the organic products were extracted with dichloromethane (2 × 20 mL). The organic fraction was washed with water (10 mL), dried over MgSO_4_ and concentrated to dryness. The crude oil was purified by silica gel chromatography using a 89/9/2 pentane/Et_2_O/NEt_3_ mixture as an eluant to furnish **7** as a pale yellow oil (0.340 g, 52 %).

*Synthesis of 5-(2-chlorobenzyl)-4,5,6,7-tetrahydrothieno[3,2-c]pyridine* [*ticlopidine* (**9**)]. To a 25 mL round-bottom flask, flushed with argon, was added acetonitrile (3 mL) and paraformaldehyde (0,17 g, 4 mmol). The resulting solution was heated at 60 °C for 2 h and the amine **3** (0.31 g, 2.2 mmol) was added. A solution of organozinc **2** (~10 mL, containing ~7.5 mmol ArZnBr) obtained following the general procedure but starting from 10 mmol of 2-bromochlorobenzene (**1**) was then added and the resulting mixture was stirred for an additional 1 h at 60 °C. A saturated ammonium chloride solution (60 mL) was added and the organic products were extracted with dichloromethane (3 × 100 mL). The organic fraction was washed with water (100 mL), dried over Na_2_SO_4_ then concentrated under reduced pressure. The remaining crude oil was purified by silica gel chromatography using a 93/5/2 pentane/CH_2_Cl_2_/NEt_3_ mixture as an eluant to furnish **9** as a pale yellow oil (0.55 g, 95 %). ^1^H-NMR: *δ* 7.54 (dd, *J* = 7.4, *J* = 1.9 Hz, 1H), 7.38 (dd, *J* = 7.6, *J* = 1.6 Hz, 1H), 7.40-7.25 (m, 2H), 7.10 (d, J = 7.5 Hz, 1H), 6.72 (d, *J* = 5,1 Hz, 1H), 3.86 (s, 2H), 3.68 (s, 2H), 2.89-2.94 (m, 4H); ^13^C-NMR: *δ* 135.9, 134.3, 133.7, 133.4, 130.7, 129.5, 128.3, 126.8, 125.3, 122.7, 58.3, 53.0, 50.7, 25.4; MS (ESI^+^): m/z = 265 (42), 264 (45), 263 (100), 262 (78), 228 (51), 125 (31), 110 (77), 89 (13).

## Conclusions

In conclusion, the results presented in this study indicate that a three-component Mannich-type reaction involving an arylzinc reagent as the nucleophile can constitute a very reliable methodology for the synthesis of commercial drugs displaying a α-amino ester or a benzylamine core. Indeed, (±)-clopidogrel (**7**) and its ethyl analogue **6**, as well as ticlopidine (**9**) were obtained in satisfactory to high yields through this practical and straightforward procedure. A current research interest concerns the application of this methodology as a key step for the preparation of a larger range of pharmaceutical drugs and synthetically useful scaffolds.

## Figures and Tables

*Conditions and yields*: i. 1. MeOH, HCOOH, 60 °C, MW; 2. THF-HCl_aq_ (9:1), r.t., 81%; ii. DMF, r.t., 49%; iii. MeOH, H_2_SO_4_, heat, 92%; iv. HCl, MeOH, reflux, 5 h, 84%; v. SOCl_2_, heat, 83%; vi. K_2_CO_3_, MDF, 90 °C, 4 h, 45%.

**Table:** 

Entry	Conditions	Time (h)	Conversion (%)^b^	Yield (%)
1	H_2_SO_4_, cat.	18	<5	-
2	H_2_SO_4_, excess	18	88	85*^c^*
3	MeONa, excess	0,5	90	86*^c^* (52*^d^*)
4	I_2_, cat.	18	<5	-
5	In + I_2_, excess	18	72	68*^c^*

*^a^* Experiments were typically conducted with 2 mL of methanol and 0.68 g (2 mmol) of **6**. *^b^* Consumption of Clopidogrel analogue **6**, monitored by GC. *^c^* GC yield. *^d^* Isolated yield.

**Table 1:** Influence of organozinc amount. *^a^*

Entry	Organozinc 2 (equiv.)*^b^*	Time (h)	Conversion (%)*^c^*	Yield (%)
1	1	1	0	-
2	2	1	0	-
3	2.5	1	<5	-
4	3	0.5	100	82*^d^* (>95^e^)
5	3.5	0.5	100	>95*^e^*
6	4	0.5	100	>95*^e^*

*^a^* Experiments were typically conducted with 7 mL of acetonitrile, 0.14 g (1 mmol) of the amine **3**, 0.5 mL (~2.5 mmol) of ethyl glyoxylate in toluene and 1.2 g (19 mmol) of zinc dust. *^b^* Relative to the amine **3**. *^c^* Consumption of the amine **3**, monitored by GC. *^d^* Isolated yield. *^e^* GC yield.

**Table 2:** Influence of glyoxylate amount. *^a^*

Entry	Glyoxylate 4 (equiv.)*^b^*	Time (h)	Conversion (%)*^c^*	Yield (%)*^d^*
1	1.25	1.5	43	-
2	2	0.5	100	78 (>95^e^)
3	2.5	0.5	100	82 (>95^e^)
4	5	0.5	100	75 (>95^e^)

*^a^* Experiments were typically conducted with 7 mL of acetonitrile, 0.7 mL (6 mmol) of 2-bromochlorobenzene, 0.14 g (1 mmol) of the amine **3**, 1.2 g (19 mmol) of zinc dust, 0.13 g (0.6 mmol) of CoBr_2_. *^b^* Relative to the amine **3**. *^c^* Consumption of the amine **3**, monitored by GC. *^d^* Isolated yield. *^e^* GC yield.

**Table 3:** Influence of the reaction temperature. *^a^*

Entry	Temperature (°C)	Time (h)	Conversion (%)*^b^*	Yield (%)
1	0	2	25	-
2	25	0.5	100	78*^c^* (>95*^d^*)
3	60	0.3	100	>95*^d^*

*^a^* Experiments were typically conducted with 7 mL of acetonitrile, 0.7 mL (6 mmol) of 2-bromochlorobenzene, 0.14 g (1 mmol) of the amine **3**, 0.4 mL (~2 mmol) of ethyl glyoxylate in toluene, 1.2 g (19 mmol) of zinc dust, 0.13 g (0.6 mmol) of CoBr_2_. *^b^* Consumption of the amine **3**, monitored by GC. *^c^* Isolated yield. *^d^* GC yield.

**Table 4:** Scale-Up. *^a^*

Entry	Amine 3 (mmol)	Time (h)	Conversion (%)*^b^*	Yield (%)*^c^*
1	1	0.5	100	78
2	5	1	100	89
3	30	1.5	100	75

*^a^* Experiments with 1 mmol of **3** were typically conducted with 7 mL of acetonitrile, 0.7 mL (6 mmol) of 2-bromochlorobenzene, 0.4 mL (~2 mmol) of ethyl glyoxylate in toluene, 1.2 g (19 mmol) of zinc dust, 0.13 g (0.6 mmol) of CoBr_2_. For scale-up, all amounts were increased proportionally to maintain similar concentrations. *^b^* Consumption of the amine **3**, monitored by GC. *^c^* Isolated yield.

**Table 5:** Transesterification of clopidogrel analogue **6***^a^* 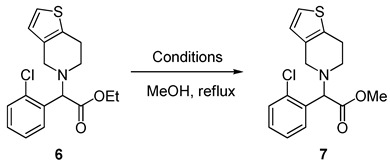

Entry	Conditions	Time (h)	Conversion (%)^b^	Yield (%)
1	H_2_SO_4_, cat.	18	<5	-
2	H_2_SO_4_, excess	18	88	85*^c^*
3	MeONa, excess	0,5	90	86*^c^* (52*^d^*)
4	I_2_, cat.	18	<5	-
5	In + I_2_, excess	18	72	68*^c^*

*^a^* Experiments were typically conducted with 2 mL of methanol and 0.68 g (2 mmol) of **6**. *^b^* Consumption of Clopidogrel analogue **6**, monitored by GC. *^c^* GC yield. *^d^* Isolated yield.
